# Dandelion (*Taraxacum mongolicum* Hand.-Mazz.) Supplementation-Enhanced Rumen Fermentation through the Interaction between Ruminal Microbiome and Metabolome

**DOI:** 10.3390/microorganisms9010083

**Published:** 2020-12-31

**Authors:** Yan Li, Mei Lv, Jiaqi Wang, Zhonghong Tian, Bo Yu, Bing Wang, Jianxin Liu, Hongyun Liu

**Affiliations:** 1 Institute of Dairy Science, MoE Key Laboratory of Molecular Animal Nutrition, College of Animal Sciences, Zhejiang University, Hangzhou 310058, China; 11717022@zju.edu.cn (Y.L.); 21917080@zju.edu.cn (M.L.); 21817080@zju.edu.cn (J.W.); liujx@zju.edu.cn (J.L.); 2 Shandong Yinxiang Weiye Group Co. Ltd., Heze 401420, China; tianye-1@163.com (Z.T.); yxwyyubo@163.com (B.Y.); 3 State Key Laboratory of Animal Nutrition, College of Animal Science and Technology, China Agricultural University, Beijing 100193, China

**Keywords:** dandelion, microbiome, metabolome, rumen fermentation, dairy cows

## Abstract

This study investigated the effects of dandelion on the ruminal metabolome and microbiome in lactating dairy cows. A total of 12 mid-lactation dairy cows were selected and randomly classified into two groups, supplementing dandelion with 0 (CON) and 200 g/d per cow (DAN) above basal diet, respectively. Rumen fluid samples were collected in the last week of the trial for microbiome and metabolome analysis. The results showed that supplementation of DAN increased the concentrations of ammonia nitrogen, acetate, and butyrate significantly. The rumen bacterial community was significantly changed in the DAN group, with Bacterioidetes, Firmicutes, and Proteobacteria being the main ruminal bacterial phyla. The abundance of *Ruminococcaceae_NK4A214_group*, *UCG_005*, and *Christensenellaceae_R_7_group* were relatively higher, whereas that of *Erysipelotrichaceae_UCG_002* and *Dialister* were lower in the DAN than those in the CON. Metabolomics analysis showed that the content of d-glucose, serotonin, ribulose-5-phosphate, and d-glycerate were higher in the DAN group. These metabolites were enriched in the starch and sucrose metabolism, pentose phosphate pathway, tryptophan metabolism, and glycerolipid metabolism. The ribulose-5-phosphate and d-glycerate were correlated with *Ruminococcaceae_NK4A214_group*, *UCG_005*, *and Christensenellaceae_R-7_group* positively. This study demonstrated that the supplementation of dandelion impacts the ruminal microorganisms and metabolites in a way that rumen fermentation was enhanced in lactating dairy cows.

## 1. Introduction

Dandelion is a member of genus *Taraxacum*, which is distributed in the warmer temperate zones of the northern hemisphere widely [1]. As an edible medicinal herb, this genus is primarily used as an anti-inflammatory, choleretic, antibacterial, anti-allergy, and antioxidant owing to its bioactive metabolites such as phenolic compounds, sesquiterpene lactones, polysaccharide, and flavonoids [2]. These metabolites derived from medicinal plants have been regarded as potential substitutes for traditional antibiotics in the raising of dairy cows [3]. Moreover, the usage of medicinal plants and their bioactive metabolites in domestic animals has been a popular direction in animal research and application through the food chains [4,5].

Medicinal herbs and their extracts potentially affect the ruminal microbial community and exert a far-reaching influence on rumen fermentation, animal production, and health status [6]. The enhanced profile of rumen fermentation provided more nutrients as substrates for milk production. However, most supplements were designed to be protected instead of degrading in the rumen due to the digestibility of rumen fermentation. Medicinal herbs could exert different beneficial functions because of the bioactive compounds generated in the rumen [2]. Hence, it is meaningful to develop medicinal herbs as new supplements compared to the rumen-protected additions. For instance, Sun et al. [7] revealed that *Perilla frutescens* leaf supplements could increase the milk yield and induce alterations in the bacterial composition in early-lactation of Holstein dairy cows. Elsewhere, the addition of saponins increased the abundance of *Prevotella_1*, *Prevotellaceae_YAB2003*, and *Ruminococcaceae_UCG_002*, which was correlated with rumen fiber digestion and nutrient digestion in dairy cattle [8]. Consequently, the ruminal microbiome has close relationships with the development and growth of host animals [9]. Moreover, research on rumen metabolism and microbial ecology is essential to study the interactions between the host, substrates available, end products of digestion, and microorganisms [10]. However, the relationships between ruminal microbiota and metabolites are not clearly elucidated. Therefore, this study aimed to investigate the effect of dandelion on ruminal microbiome and metabolome in lactating dairy cows.

## 2. Materials and Methods

### 2.1. Animals, Diet, and Experimental Design

Thirty primiparous mid-lactation dairy cows were used in the animal feeding experiment. A total of 12 primiparous mid-lactation dairy cows were selected, blocked, and randomly categorized into two treatment groups with 6 cows in each group for this study. The critical selection was based on the average value of milk production from either of the two groups, respectively. Cows were fed the same basal diet with dandelion supplementation of 0 (CON) and 200 (DAN) g/d per cow, respectively. The ingredients and nutrient composition of total mixed ration (TMR) are presented in Table 1. All cows were raised in a sand-bedded barn and fed ad libitum with free access to clean water. Feeding and milking occurred 3 times per day (0730, 1430, and 2130 h). The formal experiment lasted for 8 weeks with an extra 10 days’ adaptation period. Experimental animals were provided by the dairy farm owned by Yinxiang Weiye Group Co., Ltd. (Shandong, China). All procedures of this experiment were confirmed and approved by the Animal Care Committee of Zhejiang University (Hangzhou, China).

### 2.2. Samples Collection and Measurement

An oral stomach tube was used to collect the rumen fluid 3 h after morning feeding on the last day of the last week of the experiment [11]. The initial 150 mL rumen fluid was discarded and the pH value was immediately measured. One sample was collected from each cow, and every sample was divided into 3 tubes, one was put into liquid nitrogen immediately and waited for DNA extraction, whereas others were used to determine the ammonia nitrogen (NH_3_-N) and volatile fatty acids (VFA) [12,13].

### 2.3. Bioactive Compounds Certification of Dandelion

The DAN was characterized by the following methods chemically: 20 mg sample was weighted into an EP tube accurately, and 1 mL extract solution (water:methanol:acetonitrile = 1:2:2) containing 1 μg/mL internal standard was added. The samples were homogenized at 35 Hz for 4 min and sonicated for 5 min with a 3 times repetition in an ice-water bath. Then all samples were incubated for 1 h and centrifuged at 12,000 rpm for 15 min. The last supernatant fluid was transferred into a fresh glass vial for LC/MS analysis. The analysis was performed by a UHPLC system (Agilent Technologies, Santa Clara, CA, USA) with a UPLC HSS T3 column connected to a QE mass spectrometer (Santa Clara, CA, USA). The injection volume was 2 μL. The QE mass spectrometer was used because it can acquire MS/MS spectra in information-dependent acquisition (IDA) mode under the control of the acquisition software (Xcalibur 4.0.27, Thermo, Waltham, MA, USA). In this mode, the acquisition software continuously evaluates the full scan MS spectrum. The ESI source conditions were set as follows: Aux gas flow rate was 15Arb, sheath gas flow rate was 45 Arb, capillary temperature was 400 °C, MS/MS resolution was 17,500, full MS resolution was 70,000, spray voltage was 4.0 kV (positive) or −3.6 kV (negative), and collision energy was 20/40/60 in NCE mode, respectively.

### 2.4. DNA Extraction and Sequencing

According to the instructions of the manufacturer, the genomic DNA of microorganisms was extracted from rumen fluid by E.Z.N.A.^®^ soil DNA Kit (Omega Bio-Tek, Norcross, GA, USA). The DNA extract was checked and approved on 1% agarose gel, whereas DNA purity was measured using NanoDrop 2000 UV-vis spectrophotometer (Thermo, MA, USA). The V3-V4 hypervariable region of the bacterial 16S rRNA was amplified with primer pairs 806R (5′-GGACTACHVGGGTWTCTAAT-3′) and 338F (5′-ACTCCTACGGGAGGCAGCAG-3′) by an ABI PCR thermocycler (GeneAmp^®^ 9700, ABI, Vernon, CA, USA). The PCR mixtures included 2.5 mM dNTPs 2 μL, reverse primer (5 μM) 0.8 μL, forward primer (5 μM) 0.8 μL, template DNA 10 ng, 5 × TransStart FastPfu buffer 4 μL, TransStart FastPfu DNA Polymerase 0.4 μL, and finally ddH_2_O up to 20 μL. The resulting PCR product was extracted from 2% agarose gel, purified and quantified by the AxyPrep DNA Gel Extraction Kit (Axygen, Union City, CA, USA) and Quantus™ Fluorometer (Promega, Madison, WI, USA) respectively. All purified amplicons were pooled in equimolar and paired-end sequenced (2 × 300) on an Illumina MiSeq platform on the basis of the protocols of Shanghai Majorbio Bio-Pharm Technology Co. Ltd. (Shanghai, China). All of the raw reads were uploaded into the database of the NCBI Sequence Read Archive (SRA) (Accession Number: SRP266318).

The raw 16S rRNA gene sequencing reads were demultiplexed, quality-filtered by Trimmomatic and merged by FLASH with the following criteria: (i) the 300 bp reads were truncated at any site receiving an average quality score of <20 over a 50 bp sliding window, and the truncated reads shorter than 50 bp were discarded, reads containing ambiguous characters were also discarded; (ii) only overlapping sequences longer than 10 bp were assembled according to their overlapped sequence. The maximum mismatch ratio of overlap region is 0.2. Reads that could not be assembled were discarded; (iii) Samples were distinguished according to the barcode and primers, and the sequence direction was adjusted, exact barcode matching, 2 nucleotides mismatch in primer matching.

OTUs with 97% similarity cutoff were clustered by UPARSE (version 7.1, http://drive5.com/uparse/), and the chimeric sequences were identified and eliminated. The RDP classifier (http://rdp.cme.msu.edu/) passed a confidence threshold of 0.7 and analyzed the classification of each OTU representative sequence against the 16S rRNA Silva 138 database (https://www.arb-silva.de/browser/). The LEfSe was used to determine the most reliable bacteria in explaining the differences between CON and DAN by combining a nonparametric factorial Kruskal–Wallis (KW) sum-rank test for statistical significance with additional tests to assess the relevant effects and biological consistency. Bacteria with LDA scores (>2.5) were selected to exhibit the differential abundance between the CON and DAN groups. For the expression of rumen bacterial genera, “Unclassified bacteria” means no classification information corresponding to the sequence was found in the database, “norank bacteria” means there is no clear classification information or classification name at a certain taxonomic level.

### 2.5. Metabolomics Analysis

We weighed a 100 μL ruminal fluid sample, and the metabolites were extracted by a 500 µL water: methanol (1:4, *v*/*v*) solution which contained 2% l-2-chlorophenyl alanine. The mixture was homogenized at 50 Hz for 3 min at −10 °C. 200 μL chloroform was added and then homogenized at 50 Hz for 3 min at −10 °C. The mixture was given ultrasound treatment at 40 kHz for 10 min at 5 °C after vortexing for 30 s. This step was repeated 3 times. The sample was allowed to settle for 30 min, and then centrifuged at 12,000× *g* at 4 °C for 20 min, and the resulting supernatants were carefully transferred to a fresh glass bottle and vacuum-dried. After 80 μL methoxy amine hydrochloride was added, the samples were shaken for 2 min and incubated for 90 min at 37 °C to carry out an oximation reaction. For derivatization, 80 μL bis(trimethylsilyl) trifuoroacetamide (BSTFA) reagent which contained 20 μL n-hexane and 1% trimethylchlorosilane (TMCS) was added and placed for 60 min at 70 °C after shaken for 2 min. All samples were kept for 30 min at 25 °C and waited for detection by GC-MS.

The detection was performed by an Agilent 8890B gas chromatography with an Agilent 5977B mass selective detector, inert electron impact ionization (EI) source, and 70 eV ionization voltage (Agilent, Santa Clara, CA, USA). Analyte compounds were isolated from a HP-5MS capillary column (30 m × 0.25 mm × 0.25 μm), using 99.999% helium as a carrier gas at a constant flow rate (1 mL/min). The temperature of the GC column was programmed to hold at 60 °C, then rise to 310 °C at a rate of 8 °C per minute and hold for 6 min at the final temperature. The injection volume of derivatized samples was 1 µL and introduced in splitless mode with the inlet temperature of 260 °C. The ion sources temperature was 230 °C and the quadrupole temperature was 270 °C. Data acquisition was conducted on full scan mode with a range of 50–500 m/z. The original metabolite data were uploaded into the database of MetaboLights (No. MTBLS2342).

### 2.6. Correlation and Statistical Analysis

The ruminal fermentation parameters were analyzed by the software of SAS (version 9.2, mixed model). Results were shown as least squares mean which were calculated and separated by the PDIFF option in SAS. The result significance was declared at *p* ≤ 0.05, trends were declared at 0.05 < *p* ≤ 0.10.

The partial least squares discriminant analysis (PLS-DA), principal component analysis (PCA), and metabolic pathways spreading and enrichment analysis were analyzed by MetaboAnalyst 4.0 (https://www.metaboanalyst.ca/). The permutating test was used to prove the effectiveness of our model. R^2^X and R^2^Y indicate the cumulative interpretation rate, Q^2^ indicates the predictive ability of the model, Q^2^ > 0.5 indicates the predictive ability of the model is better.

Correlation analyses between ruminal fermentation parameters and the top 20 genera of microbiota were calculated using Spearman’s correlation test, and connections with *p* < 0.05 and *r* > 0.54 were retained. The correlations analysis between differential metabolites and the top 20 genera of microbiota were used the same method as well.

## 3. Results

### 3.1. Chemical Composition of Dandelion

Fifty main bioactive compounds were identified in DAN by LC/MS (Table 2). We identified the top 10 bioactive compounds including β-d-glucopyranoside, caffeic acid, 7,8-dihydroxyflavone, luteolin-4’-*O*-glucoside, rutin, 9Z, 11E-linoleic acid, choline, proline, trigonelline HCl, malic acid. These compounds are mostly triterpenoids, flavonoids, alkaloids, and amino acids.

### 3.2. Rumen Fermentation Characteristics 

The concentrations of NH_3_-N, acetate, and butyrate were significantly higher in the DAN group than in the CON group (*p* < 0.05). The VFA (*p* = 0.09), isobutyrate (*p* = 0.09), isovalerate (*p* = 0.07), and the ratio of acetate to propionate (AA/PA, *p* = 0.08) tended to be higher in the DAN group (Table 3).

### 3.3. Changes in Ruminal Bacterial Communities

**Figure 1:**
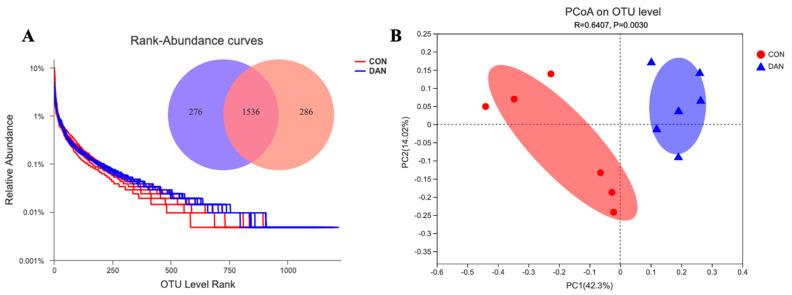
The rank-abundance curves are derived from the microbial OTU level, the Venn graph illustrating overlap of microbial OTUs among treatments at the 3% dissimilarity level (**A**). PCoA analysis of taxonomical classifications in the cows fed control (CON) and dandelion (DAN) diets (**B**).

**Figure 2:**
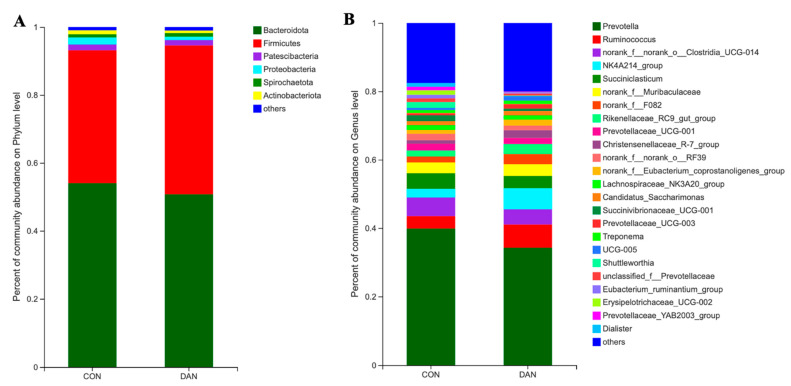
The distribution of rumen fluid bacterial taxa under phylum (**A**) and genus (**B**) levels between the treatments.

A total of 2098 OTUs were identified in these two groups, out of which 1536 OTUs were determined in both groups (Figure 1A). The DAN group had more OTUs, compared with the CON group (Figure 1A). Based on the PCoA graph (Figure 1B), the clouds derived from the CON and the DAN data were separated from each other. A higher level of species richness existed in the DAN group based on the Sobs (*p* = 0.06), Ace (*p* = 0.07), and Chao (*p* = 0.02), and a similar level in Shannon, Simpson, and Coverage, implying a changed alpha diversity in the DAN group (Table 4). Besides, 6 bacterial phyla (Firmicutes, Bacteroidetes, Actinobateriota, Proteobacteria, Spirochaetes, and Patescibacteria) were identified in rumen fluid which exhibited relatively high abundances (>1%) (Figure 2A). At the genus level, a total of 288 bacterial taxa were identified, and 27 genera had relatively higher abundances (>1%, Figure 2B). The percentage of unclassified sequences was 8.3% (24/288).

**Figure 3:**
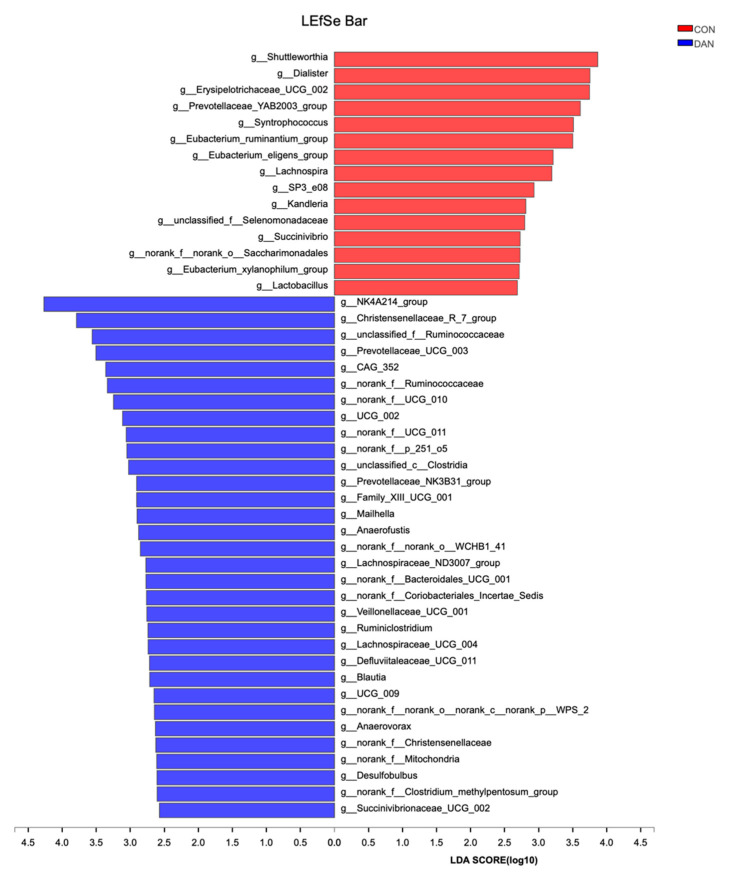
The rumen fluid bacteria showing the different abundance of values between the dandelion (DAN) and control (CON) groups. Red and blue bars represent the bacterial community of DAN with a significantly lower and higher abundance compared with that in the CON group, respectively.

We identified 47 clades as biomarkers, including the DAN and CON groups (Figure 3). Notably, 32 clades were significantly abundant in DAN group, among them, at the phylum level, 5 genus belonging to the Bacteroidota (*norank_f__Bacteroidales_UCG_001*, *norank_f__p_251_o5*, *Prevotellaceae_NK3B31_group*, *Prevotellaceae_UCG_003*, *unclassified_c__Bacteroidia*), 3 genera belonging to Desulfobacterota (*Desulfobulbus*, *Mailhella*, *norank_f__norank_o__Bradymonadales*), 21 genera belonging to Firmicutes (*Defluviitaleaceae_UCG_011*, *Christensenellaceae_R_7_group*, *Anaerofustis*, *Anaerorhabdus_furcosa_group*, *norank_f__Christensenellaceae*, *Blautia*, *Lachnospiraceae_ND3007_group*, *Lachnospiraceae_UCG_004*, *Ruminiclostridium*, *UCG_009*, *norank_f__Clostridium_methylpentosum_group*, *NK4A214_group*, *UCG_002*, *CAG_352*, *norank_f__Ruminococcaceae*, *unclassified_f__Ruminococcaceae*, *norank_f__UCG_010*, *norank_f__UCG_011*, *Amnipila*, *Anaerovorax*, *Family_XIII_UCG_001*, *unclassified_c__Clostridia*, *Veillonellaceae_UCG_001*). 15 clades were more abundant in CON group, among them, at the phylum level, 11 genera belonging to Firmicutes (*Eubacterium_eligens_group*, *Erysipelotrichaceae_UCG_002*, *Kandleria*, *Eubacterium_ruminantium_group*, *Lactobacillus*, *Lachnospira*, *Eubacterium_xylanophilum_group*, *Shuttleworthia*, *Syntrophococcus*, *unclassified_f__Selenomonadaceae*, *Dialister*).

### 3.4. Correlation Analysis between the Ruminal Microbiome and Fermentation Parameters

**Figure 4:**
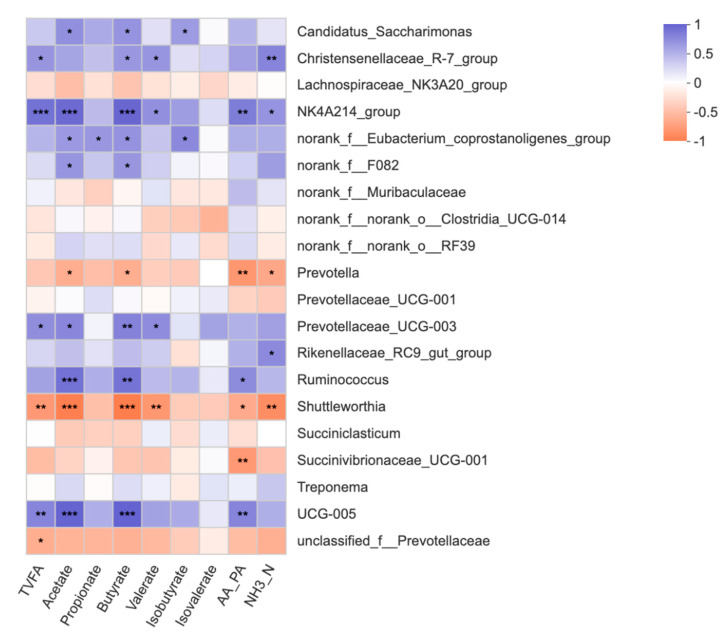
Correlation analyses between the fermentation characteristics and top 20 rumen bacterial genera. The blue squares represent the positive correlations while orange ones represent the negative correlations. * *p* < 0.05; ** *p* < 0.01; *** *p* < 0.001. NH_3_-N = ammonia nitrogen, AA/PA = the ratio of acetate to propionate, TVFA = total volatile fatty acid.

We found that, within the rumen, fermentation characteristics were correlated with the bacterial community (Figure 4). In detail, acetate, butyrate, and isobutyrate were positively correlated with *Candidatus_Saccharimonas*. TVFA, butyrate, valerate, and NH_3_-N were positively correlated with *Christensenellaceae_R-7_group*. TVFA, acetate, butyrate, valerate, AA/PA, and NH_3_-N were positively correlated with *NK4A214_group*. TVFA, acetate, butyrate, and valerate were positively correlated with *Prevotellaceae_UCG_003*. NH_3_-N was positively correlated with *Rikenellaceae_RC9_gut_group*. Acetate, butyrate, and AA/PA were positively correlated with *Ruminococcus*. TVFA, acetate, butyrate, and AA/PA were positively correlated with *UCG_005*. Acetate, butyrate, AA/PA, and NH_3_-N were negatively correlated with *Prevotella*. TVFA, acetate, butyrate, valerate, AA/PA, and NH_3_-N were negatively correlated with *Shuttleworthia*. AA/PA was negatively correlated with *Succinivibrionaceae_UCG_001*.

### 3.5. GC/MS Analysis of the Ruminal Fluid

**Figure 5:**
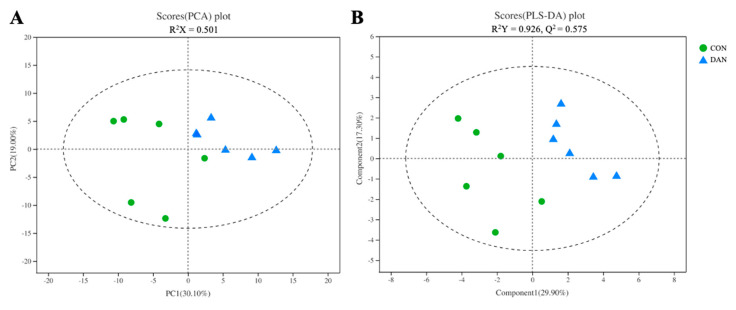
The principal component analysis (PCA) (**A**) and partial least squares discriminant analysis (PLS-DA) (**B**) score map derived from the GC-TOF/MS metabolites profiles of rumen fluid in the CON and DAN groups.

We identified 176 metabolites, including organic acids, amino acids, lipids and lipid-like molecules, amines, carbohydrates, and other metabolites. The analysis of PCA (R^2^X = 0.501) and PLS-DA (R^2^Y = 0.926; Q^2^ = 0.575) of the metabolic profiles showed a separated cluster between the CON and DAN groups (Figure 5), suggesting that the model of rumen fluid metabolomics could be used to identify the difference between the CON and DAN groups.

### 3.6. Different Metabolites between CON and DAN Groups in Rumen Fluid

In total, 36 differential metabolites were identified in CON and DAN groups (*p* < 0.05, VIP > 1). These metabolites are shown in Table 5. In general, 20 metabolites were upgraded and 16 were downgraded in the DAN group, including organic acids (palmitic acid, lactic acid, threonic acid, and phosphoric acid), sugars (d-glucose, ribulose-5-phosphate, fructose-1,6-diphosphate, and arabinose), amino acids (l-threonine and *N*-methylalanine).

### 3.7. Characterization of Metabolic Pathways

**Figure 6:**
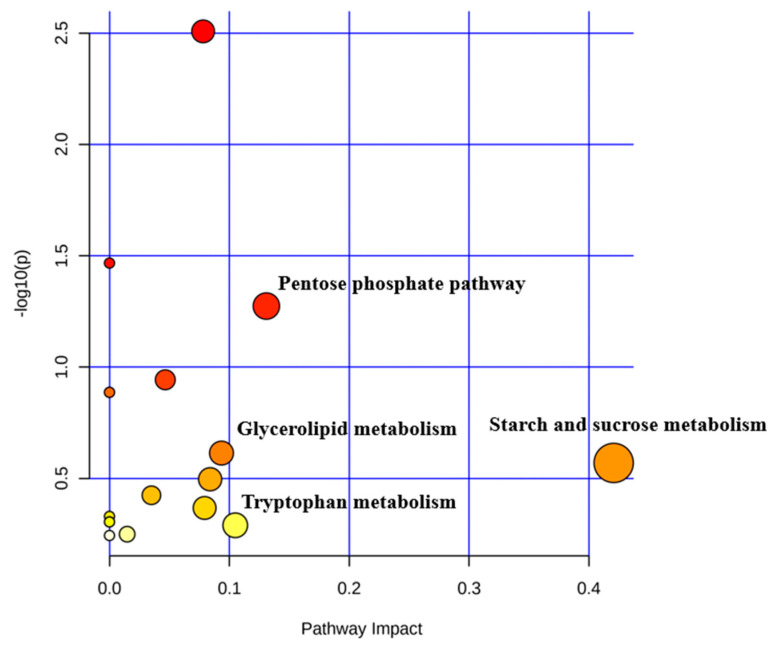
The significant metabolic pathways characterized in rumen fluid in CON and DAN group.

Fifteen metabolic pathways were obtained when all of the differential metabolites were imported into KEGG (Figure 6). Through the enrichment and pathway topology analysis, 4 pathways showed an impact value at the meaningful level: starch and sucrose metabolism (impact = 0.42), pentose phosphate pathway (impact = 0.13), tryptophan metabolism (impact = 0.10), and glycerolipid metabolism (impact = 0.09).

### 3.8. Correlation Analysis between the Ruminal Metabolome and Microbiome

**Figure 7:**
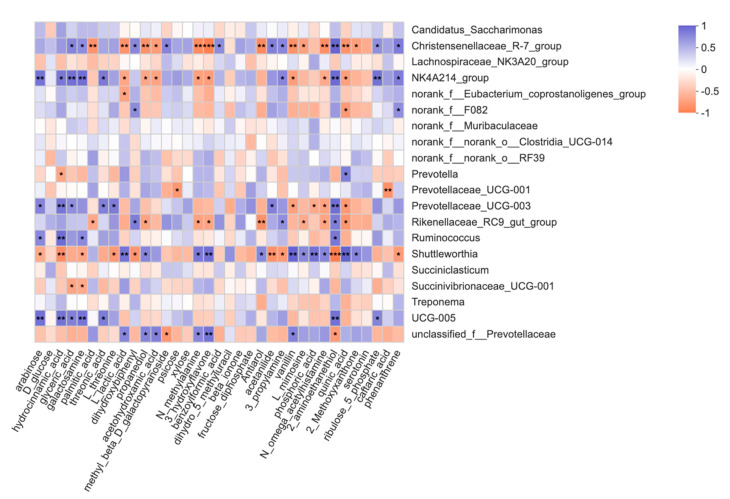
Correlation analyses between the differential metabolites and top 20 ruminal bacterial genera in both groups. The blue squares represent the positive correlations while orange ones represent the negative correlations. * *p* < 0.05, ** *p* < 0.01; *** *p* < 0.001.

Correlation analyses were constructed by the data concerning the metabolites and top 20 differential microorganisms created on Spearman correlation coefficients in CON and DAN groups (Figure 7). Significant positive correlations were found between the *UCG_005, NK4A214_group*, *Christensenellaceae_R-7_group,* and ribulose-5-phosphate; *UCG_005, Christensenellaceae_R-7_group, Prevotellaceae_UCG_003, Rikenellaceae_RC9_gut_group, Ruminococcus*, *NK4A214_group, and* 2-aminoethanethiol; *Prevotella, Shuttleworthia*, and quinic acid; *NK4A214_group, UCG_005, Ruminococcus, Prevotellaceae_UCG_003*, and arabinose. Moreover, we observed that the negative correlations were between *Christensenellaceae_R-7_group* and quinic acid; *Shuttleworthia* and 2-aminoethanethiol; *Rikenellaceae_RC9_gut_group* and antiarol.

## 4. Discussion

Dandelion improved the growth performance, energy digestibility, and feed efficiency in weaning pigs [14] and enhanced intestinal digestion and absorption in golden pompano [15]. In addition, when dairy cows were fed triterpene saponins [8] and *Perilla frutescens* leaf [7] at a specific dose, rumen fermentation characteristics were improved through regulating the rumen microbial community. In the present study, results showed that dandelion addition could improve the rumen fermentation characteristics of dairy cows. Considering the findings of the medicinal herbs used, we speculated that the supplementation of DAN could improve rumen fermentation by modifying the bacterial composition and ruminal metabolome.

Dandelion mainly contains bioactive compounds including sesquiterpene lactones, phenolic compounds, polysaccharide, and flavonoids [2], which is in accordance with our results. Bitter substances, among them, sesquiterpenes compounds, are known to stimulate digestion [1]. We also found that β-d-glucopyranoside, belonging to the sesquiterpenes compounds, was the most abundant bioactive compounds in dandelion. Many previous studies reported that dandelion causes increased bile production [16,17,18], which supports the folk usage of dandelion as an appetite and digestive stimulant [19].

In this study, Firmicutes, Proteobacteria, and Bacterioidetes were the 3 dominant ruminal bacterial phyla, which was in agreement with previous reports [7,20]. The abundance of Firmicutes was increased in the DAN group in the present study and this phylum is correlated with the degradation of structural polysaccharides as previously observed [21]. In humans and mice, the ratio of Bacteroidetes to Firmicutes has been shown to affect body fat and energy gaining [22,23] and similar findings have been reported in dairy cows [24]. Also, *Ruminococcaceae_NK4A214_group*, *norank_f__Ruminococcaceae*, *UCG_002, CAG_352*, *and Christensenellaceae_R_7_group*, belonging to Firmicutes, were abundant when cows were fed with DAN. Therefore, the supplementation of DAN may improve the degradation of structural polysaccharides in the rumen. On the other hand, we found that the genera *Dialister* and *Erysipelotrichaceae_UCG_002* were lower in the DAN group. Notably, Hayashi et al. [25] demonstrated that *Dialister* populations were positively correlated with hyposalivation, which could play an important function in changing the rumen buffering ability and fluid turnover. *Erysipelotrichaceae_UCG_002* belongs to the family of *Erysipelotrichaceae* and was previously reported to be associated with VFA synthesis and energy generation [26]. The specific association of *Erysipelotrichaceae_UCG_002* with VFA is still elusive, and thus requires an in-depth exploration in the future.

A positive correlation was found between *Ruminococcaceae_NK4A214_group* and butyrate, and the abundance of *Ruminococcaceae_NK4A214_group* was higher in the DAN group. Members of the *Ruminococcaceae* family were vital for the cellulose-degrading [27], which was further validated through transcriptomic analyses [28] and metagenomics [29]. Elsewhere, hypertriglyceridemia-related necrotizing pancreatitis rats were reported to exhibit a lower intestinal *Christensenellaceae_R-7_group* abundance [30]. Therefore, the increased *Christensenellaceae_R-7_group* abundance in the DAN group confirmed dandelion as a beneficial supplement in improving the rumen environment. Moreover, *Rikenellaceae_RC9_gut_group* was elevated, and it was reported that this genus was closely correlated with members of the *Alistipes* genus within the *Rikenellaceae* family [31], which was involved in the degradation of structural carbohydrates [21,32,33]. Therefore, it is worth noting that the degradation of nutrients in the rumen could be enhanced by the specific bioactive compounds in the DAN group.

Metabolomics analysis revealed that the levels of d-glucose, serotonin, ribulose-5-phosphate, and d-glycerate were higher in the DAN group. d-glucose is an important ending substance of starch and sucrose metabolism. It could be used as the raw material in the glycolytic pathway and enter the TCA cycle. This pathway plays an important role in energy supply and cellular respiration to all cells [34], which is vital to the in vivo metabolic efficiency and production performance of dairy cows [35]. Ribulose-5-phosphate and d-glycerate are the important substances of the pentose phosphate pathway (PPP), which is the first step in glucose metabolism from the glycolysis branch. Meanwhile, the PPP is the main source of NADPH, which is essential for removing active oxygen and fatty acid synthesis process [36], which might supply more energy for rumen microorganism growth and help the inhibition of rumen hydrogenation. Serotonin or 5-hydroxytryptamine (5-HT) could be synthesized from tryptophan, and it plays a vital role as a short-term homeostasis regulator of milk yield in mammary glands [37]. However, it could induce the forestomach hypomotility through 5-HT_4_ receptors [38]. Thus, the decreased value of 5-HT in the DAN group suggested a relatively sufficient fermentation status in the rumen. To sum up, the changed rumen fluid metabolome indicated the enhanced rumen fermentation by DAN supplementation.

The relationship between rumen metabolome and microbiome was reported in a goat model [39]. However, whether the ruminal microorganisms can interact with the metabolites remains unclear in dairy cows. Rumen microbial carbon degradation could produce acetate, butyrate, and propionate, which are the critical short-chain fatty acids that could contribute 80% of the host’s energy [40]. Our results showed a significantly higher concentration of acetate and butyrate, suggesting a sufficient energy supply in the rumen fermentation process. Metagenome analysis has revealed that Firmicutes genomes could encode glycoside hydrolase genes for hemicellulose degradation [41]. In the present study, the correlations between the ruminal metabolome and microbiome were identified and found that the fermentation-associated phenotypes were correlated with Firmicutes bacteria positively. Rumen ribulose-5-phosphate and D-glycerate were positively correlated with the *Christensenellaceae_R-7_group*, *UCG_005*, and *Ruminococcaceae_NK4A214_group*, which belong to the Firmicutes phylum. This indicates that these metabolites may be correlated with the degrading of structure carbohydrate and starch microorganisms. Nevertheless, these results require further verification through metagenomics.

## 5. Conclusions

This study revealed that dandelion could enhance the rumen fermentation by improving the abundance of rumen Firmicutes bacteria and the ruminal metabolic pathway that contributes to the application of dandelion as a potential functional feed additive for lactating dairy cows. In the future, it could be possible to reveal the functional microbial with dandelion through metagenomics and develop other similar medicinal herbs in dairy research.

## Figures and Tables

**Table 1:** Ingredient and nutrient composition of total mixed ration (TMR).

Ingredient	% of DM ^1^	Chemical Composition	% of DM
Corn silage	30.36	CP	16.68
Alfalfa hay	13.68	NDF	37.89
Corn grain	12.70	ADF	19.81
Orts hay	3.26	EE	2.92
Cotton seed	3.26	Ash	7.22
Beet pulp	5.54	NE_L_, Mcal/kg of DM	1.68
Soybean meal	9.12		
DDGS ^2^	4.33		
Flaked corn	9.77		
Rapeseed meal	2.93		
Extruded soybean	1.63		
Concentrate ^3^	3.42		

^1^ DM: dry matter; ^2^ DDGS: distillers dried grains with solubles; ^3^ Concentrate includes: 28.68% fat powder, 10.52% Ca (HCO_3_)_2_, 19.12% Na_2_HCO_3_, 9.56% stone powder, 11.47% salt, 1.53% rumen protected methionine, 4.78% yeast, 4.78% yeast culture, 4.78% MgO, 4.78% premix. Premix includes (per kg of DM): 73 KIU of Vitamin A, 1200 IU of Vitamin E, 17 KIU of Vitamin D, 20 mg of Se, 255 mg of Cu, 60 mg of Co, 40 mg of Fe, 1635 mg of Zn, 40 mg of I, 708 mg of Mn.

**Table 2:** The relative proportion of main bioactive compounds (% of total abundance of bioactive compounds) in dandelion (DAN) using LC/MS.

Bioactive Compounds	Proportion, %	Bioactive Compounds	Proportion, %
β-d-Glucopyranoside	12.32	Myristic acid	0.42
Caffeic acid	9.57	Tartaric acid	0.38
7,8-Dihydroxyflavone	8.61	Methylsuccinic acid	0.38
Luteolin-4’-*O*-glucoside	7.96	Methyl palmitoleate	0.27
Rutin	7.62	Heptadecanoic acid	0.16
9Z,11E-Linoleic acid	5.89	L-Valine	0.11
Choline	5.60	2-Hexenal	0.09
Proline	5.36	Palmitic acid	0.08
Trigonelline HCl	4.11	Piperidine	0.08
Malic acid	3.88	l-Phenylalanine	0.07
Valine	3.78	Sucrose	0.05
Trans-Vaccenic acid	3.32	l-Glutamic Acid	0.04
Phenylalanine	2.94	Chlorogenic acid	0.04
Methyl vanillate	2.65	Aspartate	0.04
Quercetin	2.58	Nicotinamide	0.04
Stachydrine	1.75	Dehydrocostus lactone	0.04
l-Isoleucine	1.63	Phthalic anhydride	0.04
9-Trans-Palmitelaidic acid	1.54	l-Carnitine	0.04
d-Gluconic acid	1.53	Isoimperatorin	0.03
Citrate	1.28	Tyrosine	0.03
Methyl Heptadecanoic acid	0.98	Formononetine	0.03
Mannitol	0.86	l-Theanine	0.02
Adenosine	0.64	Sarracenin	0.02
Nicotinic acid	0.55	p-Coumaraldehyde	0.01
l-Tryptophan	0.53	Decanoic acid	0.01

**Table 3:** Effects of dandelion supplementation on ruminal fermentation characteristics in mid-lactation dairy cows.

Items	Treatments		SEM	*p*-Value
	CON	DAN		
pH	6.85	6.73	0.010	0.92
Total VFA (mM)	93.78	99.65	1.982	0.09
Acetate (mM)	60.08	68.23	1.294	0.04
Propionate (mM)	19.10	18.93	0.812	0.85
Butyrate (mM)	7.16	9.24	0.237	0.05
Valerate (mM)	1.79	1.85	0.051	0.39
Isobutyrate (mM)	0.62	0.69	0.042	0.09
Isovalerate (mM)	1.59	1.66	0.026	0.08
NH_3_-N (mg/dL)	10.74	14.30	0.841	0.03
AA/PA ^1^	3.14	3.60	0.103	0.07

^1^ AA/PA = acetate: propionate.

**Table 4:** Alpha diversity of ruminal bacteria community in mid-lactation dairy cows fed control (CON) and dandelion (DAN) diets.

Items	Treatments		SEM	*p*-Value
	CON	DAN		
Sobs	1039	1156	31.1	0.06
Ace	1313	1424	31.4	0.07
Chao	1321	1454	30.6	0.02
Shannon	5.40	5.66	0.089	0.16
Simpson	0.01	0.01	0.001	0.15
Coverage	0.99	0.98	0.001	0.25

**Table 5:** Certification of significantly different metabolites in rumen fluid between the CON and DAN groups.

Metabolites	Similarity	VIP ^1^	FC ^2^	*p*-Value
Ribulose-5-phosphate	99.40	1.66	1.96	0.02
Glycerate	99.00	1.30	1.74	0.05
d-glucose	99.90	1.30	1.72	0.02
3-(Methylthio)-propylamine	72.60	2.54	1.11	<0.01
Trans caftaric acid	62.10	2.26	1.09	0.01
Psicose	91.50	2.24	1.08	0.01
β-ionone	78.90	1.91	1.07	0.01
Acetanilide	73.90	1.76	1.06	<0.01
Galactosamine	98.90	1.65	1.05	0.01
Benzoylformic acid	80.40	1.40	1.05	0.04
Threonic acid	98.60	1.45	1.04	0.02
Xylose	88.80	1.35	1.04	0.04
Phenanthrene	61.10	1.26	1.04	0.02
Arabinose	99.90	1.40	1.03	0.04
l-threonine	98.10	1.48	1.03	<0.01
Methyl-β-d-galactopyranoside	95.20	1.39	1.03	0.04
Fructose-1,6-diphosphate	77.90	1.11	1.03	0.04
Hydrocinnamic acid	99.30	1.13	1.02	0.01
2,3-Dihydroxybiphenyl	96.10	1.30	1.02	0.03
2-Aminoethanethiol	68.20	1.04	1.02	<0.01
Palmitic acid	98.80	1.00	0.99	0.03
Antiarol	74.50	1.22	0.98	0.01
l-(+) lactic acid	98.00	1.44	0.97	<0.01
1,3-Propanediol	95.90	1.67	0.97	<0.01
2-Methoxyxanthone	64.50	1.31	0.97	<0.01
3-Hydroxyflavone	82.20	1.77	0.96	<0.01
Vanillin	70.50	1.34	0.96	<0.01
l-mimosine	69.50	1.47	0.96	0.01
*N*-methylalanine	88.70	1.82	0.95	<0.01
5,6-Dihydro-5-methyluracil	79.90	1.67	0.95	0.03
Acetohydroxamic acid	95.60	1.94	0.94	0.01
Quinic acid	68.00	1.77	0.94	0.01
*N*-omega-acetylhistamine	68.50	1.92	0.93	<0.01
Phosphoric acid	69.20	2.14	0.92	0.01
Serotonin	98.40	1.40	0.67	0.01

^1^ VIP = variable importance in projection. ^2^ FC = fold change, mean value of peak area (DAN/CON).

## Data Availability

The data presented in this study are openly available in NCBI Sequence Read Archive (SRA) (Accession Number: SRP266318) and MetaboLights (No. MTBLS2342).
